# Purification, Partial Characterization, and Evaluation of the Antiulcer Activity of *Calotropis procera* Leaf Lectin

**DOI:** 10.2174/0929866529666220803162457

**Published:** 2022

**Authors:** Saed A. Al-Thobaiti, Emadeldin Hassan E. Konozy

**Affiliations:** 1 Department of Biology, Turabah University College, Taif University, Taif, Saudi Arabia;; 2 Biotechnology Department, Africa City of Technology, Khartoum, Sudan

**Keywords:** *Calotropis procera*, medicinal plant, lectin, purification, ulcer, gastroprotective

## Abstract

***Background*:** Lectins are proteins with therapeutic and diagnostic potential that can be applied in battling various ailments.

***Aim and Objective*:** This study was designed to purify and characterize the hemagglutinating activity derived from the leaves of *Calotropis procera* and its possible role in protecting the stomach against ethanol-induced lesions.

***Methods*:** The *Calotropis procera* leaf lectin (ProLec), was isolated by homogenization of the defatted leaf powder in Phosphate-Buffered Saline (PBS) and purified by affinity chromatography on Sephadex G-100. The lectin was eluted from the affinity column by 3% acetic acid and was physicochemically characterized. In a dose-dependent manner, ProLec was administered to rats with ethanol-induced ulcers, and biochemical, histopathological, and toxicological examinations were performed.

***Results*:** ProLec is a heterodimer of 75 and 68 kDa. It agglutinated all human RBCs, whereas it showed weak interaction with animal erythrocytes. The protein was optimally active at 25 °C and was labile above this temperature. ProLec exhibited two pH optima and was a metalloprotein requiring Ca, Mn, and Ni. It contains 1.6% tryptophan residues of which about 1% is exposed and critical for lectin activity. The lectin exhibited a potent gastroprotective effect against ethanol-induced gastric lesions with no apparent toxicity to both kidneys and liver. Examination of the pH of the gastric juice of lectin-treated animals indicated a possible role of lectin in maintaining stomach acidity within the normal ranges compared to the gastric juice pH of animals that received ethanol only.

***Conclusion*:** These results may suggest that ProLec could conceivably be a good future drug for the treatment of gastric ulcers, however, extensive immunological and toxicological research remains to be done.

## 1 INTRODUCTION

Lectins are carbohydrate-binding proteins that exist in almost all living species extending from bacteria to humans. Due to their carbohydrate recognition capacities, they can trigger a variety of effects but not limited to such as; bactericidal, bacteriostatic, antiviral, anticancer, antiulcer, involvement in molecular trafficking, receptor activation, *etc.* [1, 2]. Lectins have provided scientists with a valuable biological tool in the characterization and purification of varieties of glycans and glycoconjugates [3]. To date, more than 500 plant lectins have been isolated and their distinctive features were elucidated [4], crystals of many were obtained, and their structures were solved in the presence or absence of interacting ligands [5, 6]. The genus *Calotropis,* of the family Asclepiadaceae, is a shrub native to tropical and subtropical parts of Africa and Asia [7]. The plants are used routinely by locals for the treatment of many ailments and are shown to possess antipyretic, anti-inflammatory, antidiarrheal, and are endowed with cytoprotective effects for both stomach and liver [8]. The peptic ulcer remains a common gastrointestinal illness that is affecting about 10% of the total world population [9, 10] and it develops when the shielding mechanism is devoted to protecting the stomach mucosa fails [11]. Many treatments using synthetic drug regimens have been prescribed for the disease, among which are histamine-2 receptor antagonists (ranitidine hydrochloride), anticholinergic (scopolamine methyl bromide), proton pump inhibitors (pantoprazole), *etc.*, however, all these drugs are not free of adverse side effects [12] which could be severe in some incidents [13]. In addition to these demerits, they are costly and out of reach for many poor people. Therefore, the search for new drugs with the least possible side effects or none at all is in a continuous global expansion and many plant products such as flavonoids, alkaloids, tannins, gum Arabic, *etc.* have been shown to exhibit several degrees of antiulcer activity [9, 14]. While plant proteins and peptides are proven, many years ago, to possess antibacterial and anticancer activities [1], very few have been investigated for a possible protective role against gastric ulcers [15, 16]. *Calotropis gigantea* ethanolic leaf extract at a concentration below 300 mg/kg prevented gastric hemorrhage in animals administered with absolute ethanol [8]. Whereas similar studies were performed using chloroformic and ethanolic extracts of *Calotropis procera* stem, bark, leaves, flowers, and fruits, both extracts were presented with significant gastroprotective effect [17, 18]. Although the genus *Calotropis* is known for its antiulcer effects since ancient times, and while many plant lectins have been revealed to offer variable ranges of gastroprotective effects, no report has been published on studying the possible antiulcer property of this plant’s lectin. Therefore, the present work was undertaken to isolate, purify, and explore the gastroprotective character of *C. procera* leaf lectin (ProLec) in experimental animals.

## 2 MATERIALS AND METHODS

The *Calotropis procera* plant was authenticated by a plant taxonomy expert. Leaves were washed with distilled water and left in the shade until complete dryness. All chemicals, solvents, and kits were purchased from local chemical vendors and they were of the highest quality available. Sephadex G-100 and G-75 were from Pharmacia, Upsala, Sweden.

### 2.1 Animals

Rats of either sex (100 to 110 g) were left for at least 4 days to acclimatize to animal room conditions with a standard pellet diet and water. However, a day before the assay the food was removed with free access to water.

### 2.2 Ethical Statement

The experimental protocol was following the standard international ethical guidelines for the use of animals. Ethical clearance Ref. BioMed/26/2021 was priorly obtained from the respective department.

### 2.3 Protein Estimation

Protein contents in the crude and pure lectin samples were quantified throughout by the Bradford method using BSA as the standard [19].

### 2.4 Protocol Standardization for ProLec Extraction

Shade-dried *Calotropis procera* leaves were pulverized in a coffee mill till a fine powder was obtained. The Powder was defatted with n-hexane (1g/5 mL, w/v) for 4 hrs at 4 °C with continuous stirring. The defatted powder was washed with several folds of n-hexane until a faint greenish colour product was obtained which was left overnight at room temperature until no hexane odour was smelled. The obtained hexane dried powder (HDP) was divided into four portions 1 g each and transferred into 50 mL falcon tubes, to each tube 10 mL of the respective buffer (50 mM Na-acetate, pH 5; 50 mM PBS, pH 7.5; 50 mM Tris-HCl, pH 7.5; and 50 mM Borate pH 9) was added and the powder was extracted under cold condition (4 °C) for 4 hrs. The obtained slurries were passed through cheesecloth and cell debris was re-extracted for another 4 hr under the same conditions. The resultant clear supernatants obtained after centrifugation at 5009 *g* for 30 min were used to estimate the total protein contents and hemagglutination assays (HA). The buffer which resulted in the extraction of the highest hemagglutinating activity was selected and used at different molarity strengths ranging from 20 mM to 50 mM. The best extraction molarity in terms of HA was then used throughout.

### 2.5 ProLec Purification

Six hundred milligrams of crude leaf extract were applied onto the affinity column Sephadex-G-100 (20 cm x 3 cm) that was previously equilibrated with approximately 100 mL 40 mM PBS pH 7.5. Protein was recycled at least 4 times and the column was washed with 12-15 bed volumes with 40 mM PBS pH 7.5 until the protein reading at 280 nm dropped to ≤ 0.02. Retained ProLec was eluted with 3% acetic acid prepared in 0.150 MNaCl. 3mL fractions were collected for a total of 80 mL, the pH was adjusted to neutrality by 0.2 M NaOH. The lectin sample was exhaustively dialyzed against distilled water and lyophilized to dryness.

### 2.6 Native Molecular Weight Estimation

This was done using a gel-filtration column on a Sephadex G-75. The column (20 cm x 3 cm) was previously equilibrated with 40 mM PBS pH 7.5 buffer containing 200 mM glucose. The Native molecular weight of ProLec was determined by comparing its elution volume in comparison to the standard protein markers (β-Amylase: 200 kDa; Alcohol Dehydrogenase: 150 kDa; BSA: 65 kDa; Carbonic Anhydrase: 29 kDa and Cytochrome C: 12kDa).

### 2.7 Electrophoresis

Native-PAGE at pH 8.6 was performed according to Williams and Reisfeld [20]. While the subunits' molecular weights were determined by SDS-PAGE using 12% gel as per laemmli’s system [21], 30 µg of protein were loaded and the gels were stained with coomassie-brilliant blue G-250.

### 2.8 Glycoprotein Nature of ProLec

To assess the glycoprotein nature of the purified ProLec, Anthrone method was used according to Morris, 1948 [22].

### 2.9 Hemagglutinin Assay

The hemagglutinating activity of ProLec was performed using U‐shaped 96 well‐ELISA plates. A total volume of 30 μL 2‐fold serially diluted in BPS lectin solution was incubated with 30 μL 2% erythrocyte suspension of all human blood group types (A AB, B & O) obtained from apparently healthy donors. Different animal erythrocytes namely cow, camel, goat, donkey, and horses were also used. Agglutination assays were carried out with trypsin-treated or untreated human and animal erythrocytes.

Hemagglutination unit is expressed as titre, *i.e.*, the highest lectin dilution that would still give visible hemagglutination [23].

### 2.10 Lectin Sugar Specificity

To determine the lectin sugar specificity, the competition or inhibition of hemagglutination was used. A series of 300 mM sugars of mono, di- or trisaccharide nature were added separately to the well together with serially two-fold diluted ProLec and the mixture was incubated at room temperature for an hour. 2% O blood human erythrocytes were added to the wells, mixed, and incubated again for an hour at room temperature. The hemagglutination inhibition was monitored an hour after by the naked eye.

### 2.11 Effect of Temperature on ProLec Activity

Thirty µL aliquots of lectin (1 mg/mL) were incubated in a water bath at varying temperatures ranging from 20 to 90°C (± 2°C) with an increment of 10 degrees for 30 minutes. The lectin samples were immediately cooled on ice and hemagglutination activity was reported. Lectin activity assayed at room temperature (25°C ± 2°C) served as a control of 100% activity.

### 2.12 The Thermal Stability of ProLec at its Temperature Optima

This was studied by incubating ProLec at its temperature optima for 3 hrs. An aliquot was removed every 30 minutes and the hemagglutinating activity was tested.

### 2.13 Effect of pH on Lectin Activity

The stability of lectin at different buffers of variable pH values (pH 2 to pH 11) was assayed by incubating aliquots of lectin at these buffers at room temperature for 2 hours. 0.1 N HCl or 0.1 N NaOH were used to adjust the pH of the lectin solution to neutrality, and then the hemagglutinating activity was assayed. Lectin solution in 40 mM phosphate-buffered saline pH 7.5 was used as a control of 100% activity [24].

### 2.14 Effect of Chelating Agent and Metal Ions on ProLec

Lectin solution was dialyzed exhaustively against 50 mM EDTA for 24 hours with at least two changes, the ProLec solution was then dialyzed against double‐deionizeddistilled water for two days to remove the excess EDTA. EDTA-treated lectin was incubated with 2 mM metal ions, Ferrous, Zinc, Mercury, Magnesium, Manganese, and Calcium for 2 hrs at room temperature followed by a hemagglutination assay as previously shown. EDTA untreated lectin served as a control of 100% activity [24].

### 2.15 Modification of Tryptophan Residues

This was achieved by using the tryptophan selective oxidation reagent N-bromosuccinimide (NBS). The lectin solution at approx 0.4 mg/mL was made in 20 mM acetate buffer, pH 6.0 was titrated with the freshly prepared NBS (10 mM). Aliquots of NBS reagent were added gradually to the ProLec solution in installments of 20 µL each with shaking and incubation for 10 min and then monitored for the drop in absorbance at 280 nm as well as lectin hemagglutination activity. The percentage of modified tryptophan residues was evaluated as given by Spande and Witkop [25] using the following formula:


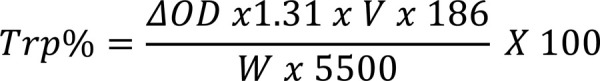


Where as:

ΔOD corrected optical density decrease at 280 nm; V: initial volume of lectin in mL; W: weight of protein titrated in mg; 5500: molar extinction coefficient of tryptophan at 280 nm; 186: molecular weight of bound tryptophan residue.

### 2.16 Antiulcer Activity of the ProLec

The animals were distributed into seven groups of six rats each (*n* = 6). Animals have interdicted from food overnight but were allowed free water access. The groups were segregated as follows:

1) Group 1 received normal saline (3 mL/kg) and served as a negative control.

2) Group 2 received absolute ethanol (3 mL/kg).

3) Group 3 received 100 mg/kg pantoprazole and served as a positive control.

4) Group 4 received 0.250 mg/kg ProLec.

5) Group 5 received 0.5 mg/kg ProLec.

6) Group 6 received 1 mg/kg ProLec.

7) Group 7 received 1 mg/kg ProLec and was used for a toxicity study.

The animals of groups 1-6 were euthanized half an hour after the treatment. The stomach was cut open in the greater curvature and ulcer scoring was done. Blood samples were taken and immediately processed for plasma. The gastric juices of the stomach were carefully withdrawn by pipetting and persevered at -20 °C until further use.

### 2.17 Toxicological Assessment

Animals of group 7^th^ were sacrificed 24 hr from the administration of 1 mg/Kg ProLec. Kidneys, liver, and blood samples were carefully obtained. Clear plasma samples were obtained after blood centrifugation at 313 *g* for 10 minutes and were used for biochemical analysis, whereas kidney and liver tissues were processed for histopathological studies.

### 2.18 Histological Examination of Gastric Lesions and Kidney and Liver Toxicological Assessment

The stomach, kidneys, and liver were fixed in a 10% saline-diluted formalin solution for histopathological analysis following the assessment of the ulcer score. The fixed stomachs were rooted in paraffin wax to obtain paraffin wax material sections. Five µm sections were stained with H and E and assessed for microscopical investigation.

### 2.19 Liver and Kidney Function Tests

Alkaline phosphatase [26], urea [27], and creatinine [28] were assessed using BioMed diagnostics^®^ kit. Whereas aspartate aminotransferase (AST/GOT) [26] and Alanine Aminotransferase (ALT/GPT) [29] were determined using BioSystem Reagents and Instruments^®^ kit. The procedures followed are exactly as per the manufacturers’ instructions.

### 2.20 Statistical Analysis

Data reported as mean ± standard deviation. The data were scrutinized by one-way ANOVA analysis of variance. The SPSS statistical package software (IBM, Chicago, IL, USA) was used. A *p*-value <0.05 was considered statistically significant.

## 3 RESULTS AND DISCUSSION

### 3.1 Standardization of Extraction Procedure and Purification of ProLec

Calotropis, a small shrub (sometimes referred to as weed), belongs to the family Apocynaceae. Calotropis is a widely distributed plant in tropical and subtropical zones, comprising two species, *procera* and *gigantea* [30]. In our routine screening of novel lectins from tropical endogenous plants, we have detected a robust hemagglutinating activity (HA) in the *Calotropis procera* crude leaves extract. Whereas no hemagglutinating activity was found in the seed extract of the plant which is contrary to Chanchadi’s work with *Calotropis gigantea* seed, who has obtained a Unit activity of up to 2^7^ with the human “O” blood group [31]. To extract a maximum lectin content, we have started our purification strategy by extracting the defatted *C. procera* leaves powder with different buffer types and molarity strengths. The best lectin content in terms of hemagglutina-ting activity was obtained with 40 mM phosphate-buffered saline pH 7.5 (Figures **1A** and **B**). Therefore, this buffer was used throughout the purification and characterization course. To test for ProLec carbohydrate specificity, 300 mM of different mono, di- and trisaccharides were used. Of which only D-glucose could successfully inhibit the hemagglu-tinating activity of ProLec. Indicating the classification of this protein as a D-glucose-binding lectin. Sephadex is a polymer of glucose molecules with α-1,6-glycosidic linkages with recurrent branchings of α-1-3 types, besides it being routinely used as gel filtraion media for protein fractionation it is also exploited as an affinity resin to purify glucose/ mannose-binding proteins [32]. Therefore, Sephadex-G 100 was used as an affinity matrix for the purification of the detected lectin. Loading of the crude extract and recycling of the effluent several times resulted in a 100% retention of the lectin in the column. The retained lectin was then eluted with 3% acetic acid prepared in 0.150 M NaCl and when the low pH of the preparation was adjusted to neutrality, all fractions which were positive for protein showed detectable hemagglutinating activity (Figure **2A**). To test the homogeneity of the preparation obtained as per Table **1** native- polyacrylamide gel electrophoresis (PAGE) was performed for the affinity eluted protein, a single band was obtained signifying the purity of the lectin (Figure **2B**). To estimate the native molecular weight of the lectin gel-filtration on Sephadex G-75 was used, to prevent the interactions of the lectin with the Sephadex G-75 resin the run was done in the presence of ProLec haptenic sugar glucose. A native molecular weight of around 147 kDa was obtained, whereas on SDS-PAGE two bands corresponding to 75 and 68 kDa were seen (Figure **2C**), indicating the heterodimeric nature of the protein. At this stage, the specific activity (Unit/mg) of the purified lectin was increased from 11 to 142 Units/mg. Whereas the purity fold was improved by 13 times and 48% total attained yield (Table **1**). Though this protein could successfully be purified through interaction with Sephadex, *Calotropis gigantea* seed lectin was neither inhibited by glucose (at up to 200 mM) nor by other mono, di, or trisaccharides [31]. Therefore, the author of this paper described *C. gigantea* seed lectin as agglutinin with a complex carbohydrate specificity [31]. Nevertheless, our isolate is in agreement with this author’s work in terms of the human blood group unspecificity, as none of the two lectins could discriminate between human ABO blood types. Treatment of erythrocytes with trypsin was found to enhance the sensitivity of ProLec towards the erythrocytes, indicating the role of this protease in exposing the hidden glycan receptors that are necessary for erythrocytes agglutination by this lectin. The lectin could poorly agglutinate all of the tested animal erythrocytes (Table **2**).

### 3.2 Effect of pH and Thermal Stability

Incubating the purified ProLec at different ranges of pH and subsequent test of the hemagglutinating activity resulted in apparently two activity optima at 4.5 and a broad one falls between 7.5 to 9.5, highlighting the possibility of the existence of this lectin in isoforms (Figure **3A**). ProLec, and unlike many plant lectins in general and *Calotropis gigantea* seed lectins in particular exhibited a very labile nature towards thermal inactivation, the lectin temperature activity optima was found to be around 25 °C (Figure **3B**), after which a sharp decline in the protein activity was reported. Moreover, at its temperature optima, ProLec remained active for only 2.5 hr after which complete inactivation was noticed (Figure **3C**). These results are contrary to those reported with its counterpart seed lectin from C. *gigantea* where the protein depicted high stability at around 40 °C for several days [31]. However, since this research group worked with a semipurified lectin, unlike our work, the presence of other protein contaminants (natural receptors) might have enhanced the lectin stability.

### 3.3 Glycoprotein Nature of ProLec

To test if the purified protein is a glyco- or simple protein, the Anthrone test for carbohydrate assessment was done. When Athrone solution was added to 200 µg ProLec an intense blue colour was produced indicating the glycoprotein nature of the lectin. Even though the majority of plant lectins are glycosylated [4, 23], few are nonglycosylated like concanavalin A (ConA) and Peanut (*Arachis hypogaea*) seeds lectin [33, 34].

### 3.4 The Need for Metal Ions

**Figure 4:**
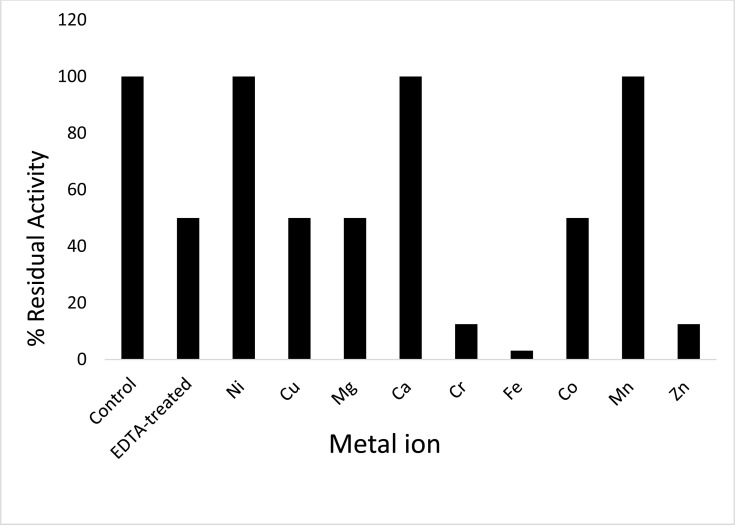
Effect of metal ion on ProLec hemagglutinating activity. 2 mg/mL lectin solution was dialyzed exhaustively against 50 mM EDTA, followed by dialyzing against double‐deionized water to remove the excess EDTA. EDTA treated lectin was incubated with 2 mM metal ions, Ferrous, Zinc, Mercury, Magnesium, Manganese, and Calcium for 2 hrs at room temperature followed by a hemagglutination assay. EDTA untreated lectin served as a control of 100% activity.

When ProLec was treated with the gelating agent EDTA, almost 50% of its original activity was abolished. However, the addition of metals like Ni^+2^, Ca^+2^ and Mn^+2^ restored the activity (Figure **4**) indicating the need for divalent metals for optimal activity. Divalent metals like Ni^+2^, Ca^+2^and Mn^+2^ are important for the optimally functioning of many plant lectins, removal of which by chelating agents makes the lectin either partially or completely inactive [24, 35, 36].

### 3.5 Modification of Tryptophan and its Importance for ProLec Activity

**Figure 5:**
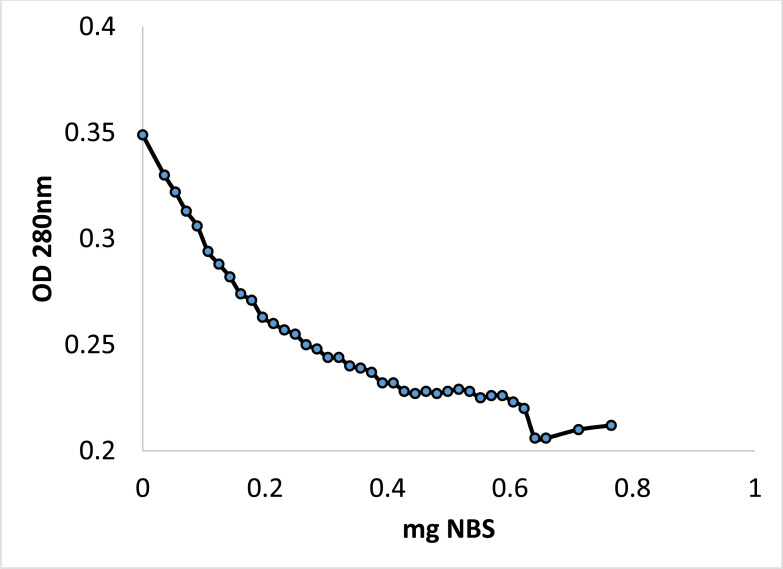
Oxidation of ProLec exposed tryptophan by N-bromosuccinimide (NBS). The ProLec solution at 0.35 mg/mL was prepared in acetate buffer, pH 5.0 was titrated with the freshly prepared NBS (10 mM). Aliquots of NBS was added gradually to the ProLec solution in instalments of 20 µL (0.035 mg) each with shaking. The drop in absorbance at 280 nm as well as the hemagglutination activity were monitored at each point. The percentage of tryptophan residues was calculated essentially as described (Spande and Witkop 1967).

Oxidation of exposed tryptophan at low pH is often done by the tryptophan-specific reagent N-bromosuccinimide (NBS) [37, 38]. Titration of ProLec with aliquots of NBS at pH 6, indicated that exposed tryptophan residues represented approx 2% of the total protein (Figure **5**), and modification of half of these tryptophans residues inactivated protein (results not shown). These results signify that these residues are essential for the sugar-binding capacity of ProLec. As could be noticed in Figure (**5**), as the tryptophan residues get modified, a noticeable drop in the absorbance at 280 nm occurs. Upon addition of approx. 0.650 mg of NBS, almost 40% of the original protein optical absorbance was lost. After which a sudden increase in the absorbance was apparent, this raising, although minor, was continued until approx 0.8 mg of NBS was added. The sudden increase in the OD after it reaches its lowest level is a common phenomenon and is attributed to the bromination of the aromatic ring of tyrosine resulting in dienone formation [25].

### 3.6 ProLec Gastroprotective Effect and the Histopathological Analysis

**Figure 6:**
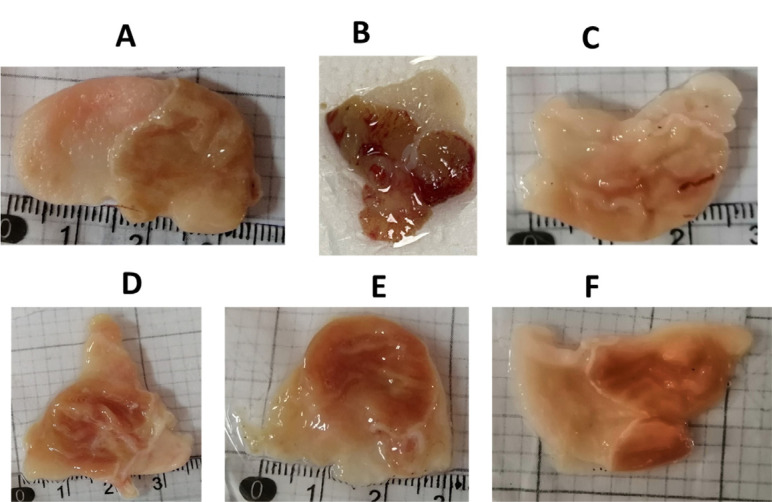
Effect of different doses of ProLec on rat’s stomach: (**A**): rat received saline; (**B**): rat received ethanol; (**C**): rat received pantoprazole; (**D**): rat received a low dose of ProLec; (**E**): rat received a medium dose of ProLec; (**F**): rat received a high dose of ProLec.

Many *Calotropis* polar and non-polar crude extracts are reported to express gastroprotective effects on experimental animals [17, 18, 39, 40]. Awaad and his colleagues have proven the effectiveness of the polar and non-polar extracts of *C. procera* in offering potent ulcerative colitis properties. As these authors didn’t purify the responsible active ingredients of the obtained antiulcer effect, they assumed it to be the flavonoids. In addition, they have confirmed the safety of the total alcoholic extract at up to 4000 mg/kg [39]. Ethanol gastric-inducedlesions are multifactorial and are linked to the reduction in the inherent stomach mucosa defensemechanism and that’s principally associated with the change in the microcirculation or maybe the oxidation stress [41]. To test for the possible cytoprotective activity of the purified ProLec, we initially performed a pilot study using a wide range of ProLec concentrations ranging from 0.25 mg to 1 mg/kg, excitingly all of these protein ranges showed, a dose-dependent, remarkable protective effect against ethanol-induced gastric lesion on experimental animals. Based on these results, we have chosen three concentrations of the lectins; 0.250 mg/kg (low dose); 0.5 mg/kg (medium dose), and 1 mg/kg (high dose) for this study. As stated, all of these concentrations produced a notable ulcer inhibition compared to control animals which received only saline (Figure **6A**-**F**). The histological section of gastric mucosa in a rat pretreated with 0.250 mg/kg ProLec exhibited intact mucosa, however, with some cellular fragmentation. Although some swelling between epithelium and submucosal tissue cells was evident, yet, no disturbance in the appearance of the epithelium tissues was spotted. Those animals who received 0.5 mg/Kg ProLec, displayed a regular mucosal layer appearance, nonetheless, some swelling was still noticeable between the epithelium and the mucosal cells with cytoplasmic vacuolization, however, both mucosa and nuclear chromatin were intact & grasped. Almost 100% protection against ethanol-induced ulcers was obtained with those animals who received 1 mg/kg. The mucosal layer and epithelium tissues remained intact with minor swelling (Figure **7A**). Both *C. procera* and *C. gigantea* are shown to demonstrate potent antioxidant potential [42] and many plant lectins have also been shown to offer varying degrees of reducing the effect of the oxidation stress [43, 44].

### 3.7 Liver and Kidney Function Tests

To further test our observation on the suitability and effectiveness of 1 mg/kg as the best obtained protective dose and to rule out any possible toxicity on the kidneys and liver tissues, we have recarried the experiment, however with extending the dosing phase from 1 hr to 24 hr. The kidney and liver function tests and histopathological analysis were then examined.

The animals were carefully monitored after the dose and until sacrification. There was no mortality sign, on the contrary, the animals maintained normal behaviour throughout and until the time of euthanization. Ultimately indicating no apparent physical toxicity. An enzyme like Alkaline phosphatase and liver and kidney biomarkers such as urea, creatinine, aspartate aminotransferase (AST/GOT), and alanine aminotransferase (ALT/GPT) were examined in the rats' serum. No statistically significant differences between the controls (rats received only saline to those who were given 1 mg/Kg ProLec) were detected (data not shown). In the histopathological studies, the liver had no ostensible mucosal disturbances, with minor cell degeneration without chromatin degeneration. A mild liver cell degeneration was noticed. The bile duct and canal and central vein persisted normal. Kidney tubules remained normal with no sign of toxicity (Figure **7B**).

The treatment of rats with purified lectin from red marine alga *Hypnea cervicornis* at 1 mg/kg for 7 days did neither affect liver and kidney weight nor showed any abnormalities in these organs’ histopathological features [45]. These outcomes may encourage us to comment on the possible safety of ProLec. However, since the lectin was administered as a single dose and the rats were sacrificed after only 24 hr of protein administration, it may be necessary to carry out a thorough toxicological study with prolonged administration of the protein using several doses. Additionally, drugs containing proteins are likely to elicit the immune system. Therefore, it may also seem necessary to perform an in-depth immunological investigation to validate any possible immune response to this protein. Thus, it will be a little early to emphasize the safety of this protein. Administration of *Calotropis procera* ethanol extract powder aqueous solution to experimental animals for five consecutive days had shown to induce ulcerative colitis healing [39]. Moreover possesses antioxidant activities [46] and triggers prostaglandin secretion [47].

### 3.8 Effect of ProLec on the Stomach’s pH

**Figure 8:**
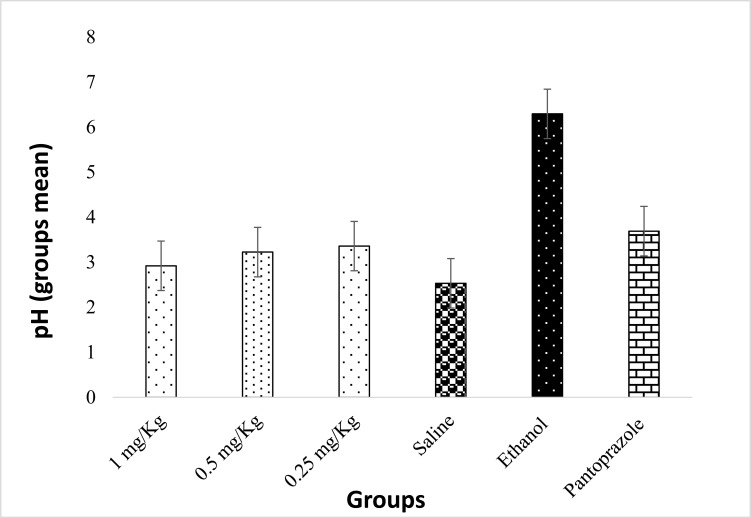
The effect of ProLec on the gastric juice pH. The values are given as mean S.E.M. (n = 6). Pantoprazole was used as a positive control. All groups treated with different doses of ProLec followed by absolute ethanol are statistically significant as compared to the group that received only absolute ethanol (*p* < 0.05).

Administration of ProLec at varying doses did not produce a significant change difference in the acidity of the animals’ gastric pH compared to the negative control (rats received saline), whereas little, insignificant raise in the pH was noticed in the gastric acidity of the positive control rats which were treated with the proton pump blocker pantoprazole. Interestingly, the rats which received solely absolute ethanol exhibited a striking significant increase in their stomachs’ pH when compared to the negative control (received saline) and to those groups which received both lectin and absolute ethanol. On the other hand, no such pH upsurge was noticed in those groups which received different ProLec doses followed by absolute ethanol (Table **3** and Figure **8**). Absolute ethanol triggers gastric lesions by producing ROS which activates lipid peroxidation in gastric tissues which would finally cause severe damage to the stomach mucosal layers [48]. Low concentrations of ethanol (≤ 5%) prompt acid secretions [49], however, higher concentrations above 40% are assumed to increase the secretion of the gastric bicarbonate through intercellular leakage, a process believed to act as an intrinsic defence mechanism mediated through Cl_2_/HCO^-^_3_ exchangers to revert the action of the elevated hydrochloric acid [50, 51]. These results excitingly emphasize a role for ProLec not only in protecting the stomach against gastric lesions but also in maintaining the stomach pH within the normal ranges.

## CONCLUSION

Although *C. gigantea* and *C. procera* are two species of the genus *Calotropis*, their purified lectins herein and in reference [31] appear physicochemically variable, signifying the gross diversity among these proteins and hence their probable physiological significance. The protection offered by the lectin against ethanol-induced lesions may be attributed to its possible action on the strengthening of the internal stomach shield through boosting gastric mucosal hemodynamics. The liver and kidneys of the treated rats showed no apparent histopathological alteration related to the control group, and biochemical parameters were within the control range of untreated animals. Moreover, by comparing the effectiveness of ProLec to pantoprazole, the standard drug for the treatment of acute ulcers, ProLec demonstrated competitive results. Based on these outcomes, the purified lectin may be considered a promising future gastroprotective drug provided that extra toxicological and immunological studies are done. Additionally understanding the precise mechanism of the protection offered by this lectin in particular and other analogous agglutinins, in general, appears tempting.

## Figures and Tables

**Figure 1A:**
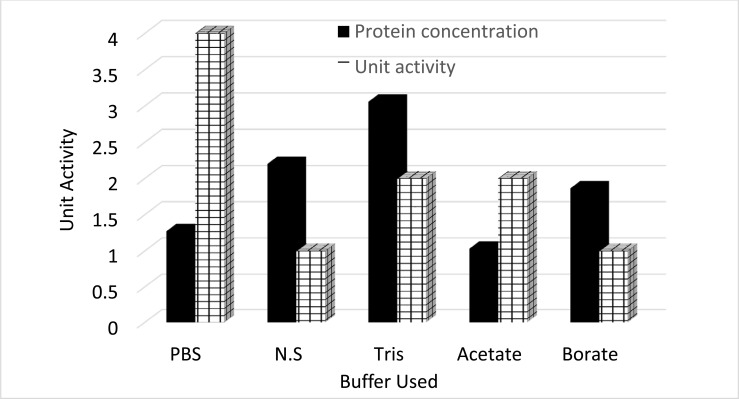
Extraction of ProLec by using different buffers. Different buffers with variable pHs were used for ProLec extraction, these buffers were: 50 mM PBS pH 7.5 (PBS); 0.145 M, NaCl (N.S); 50 mM Tris-HCl, pH 7.5 (Tris); 50 mM Na-acetate, pH 5 (Acetate); and 50 mM Borate pH 9 (Borate).

**Figure 1B:**
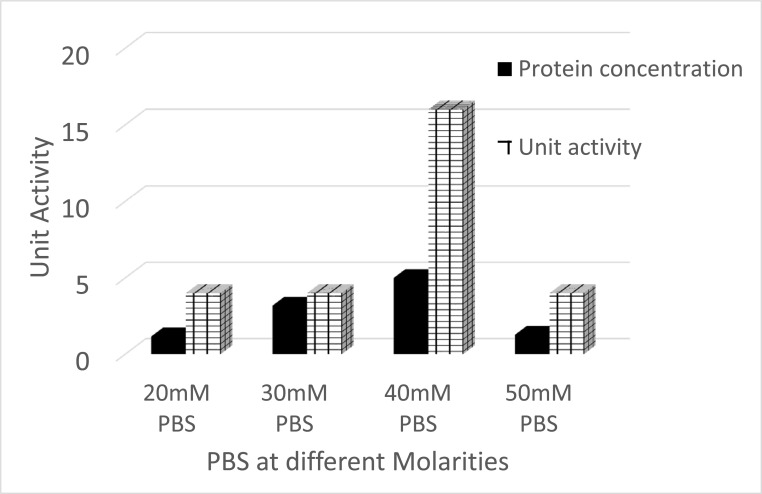
Extraction of ProLec by using PBS pH 7.5 at different molarities 20, 30, 40 and 50 mM

**Figure 2A:**
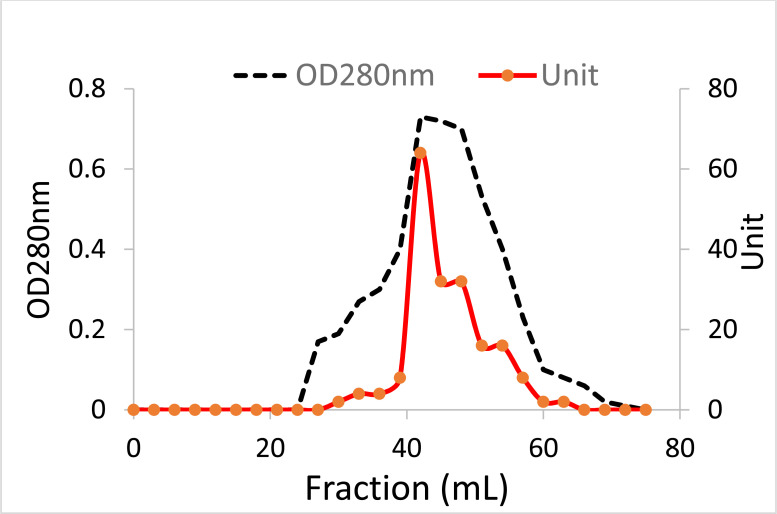
Elution of ProLec from the affinity resin Sephadex G-100. Six hundred mg of *C. procera* leaves crude extract was loaded onto the Sephadex G-100 column. The flow throw was recycled at least 3 to 4 times. The column was washed with an ample amount of 40 mM PBS pH 7.5 to remove unbound protein till the OD 280 of the washing dropped to ≤0.02. The bound lectin was eluted with 0.3% acetic acid prepared in 0.150 M NaCl. Fractions were adjusted to neutrality by 0.2 N NaOH and assayed for hemagglutinating activity.

**Figure 2B:**
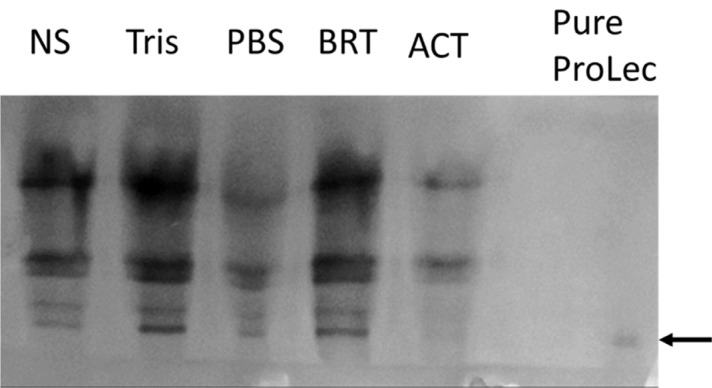
Native-PAGE crude protein extracted by varying buffers and pure ProLec obtained by affinity on Sephadex G-100. The arrow points to the pure protein. 30 µg protein were loaded and the gel was stained by coomassie brilliant blue G-250.

**Figure 2C:**
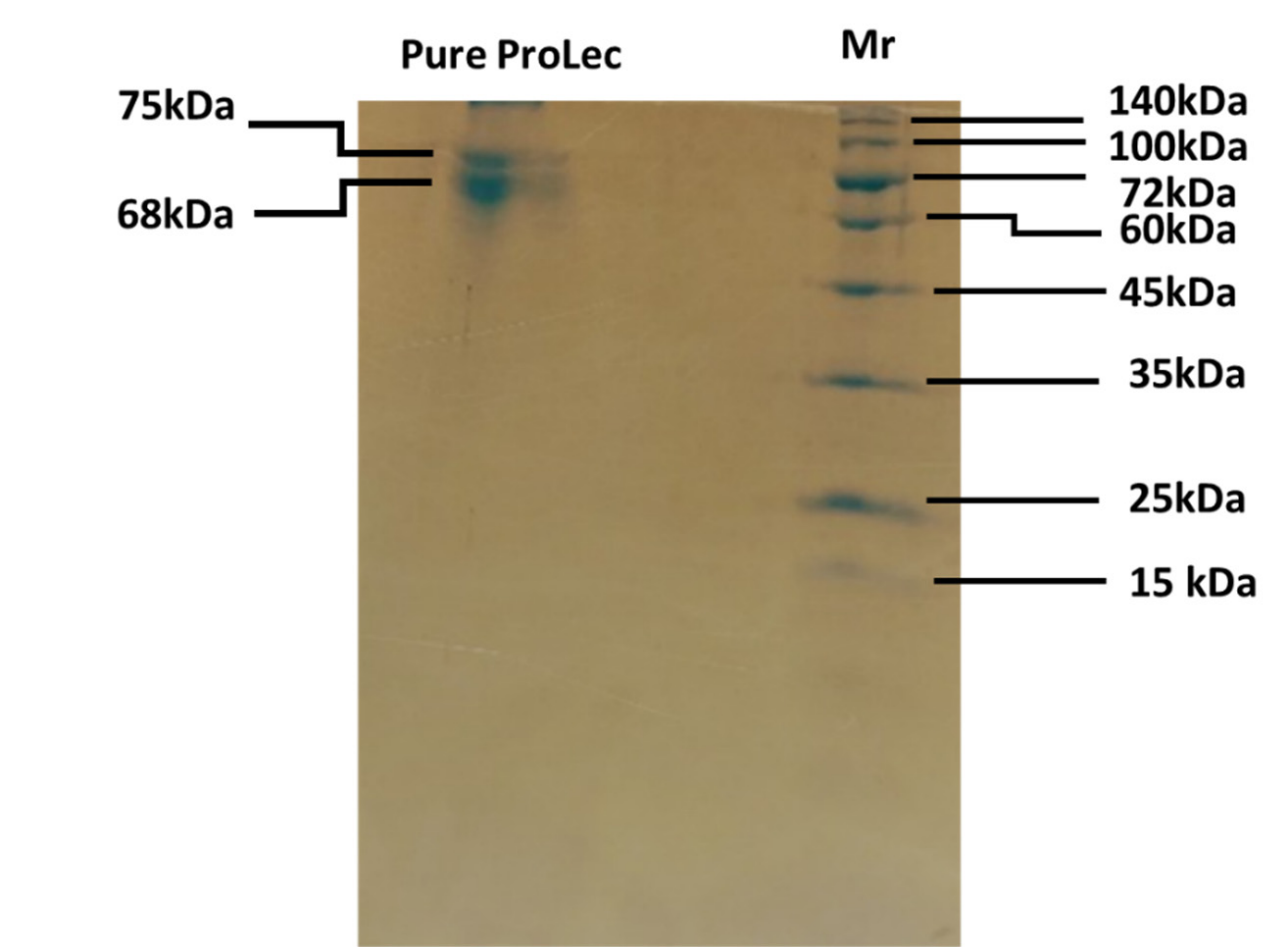
SDS-PAGE of pure ProLec obtained by affinity on Sephadex G-100. Lane on the right-hand side indicates the standard molecular weight markers 30 µg protein were loaded and the gel was stained by coomassie brilliant blue G-250.

**Figure 3A:**
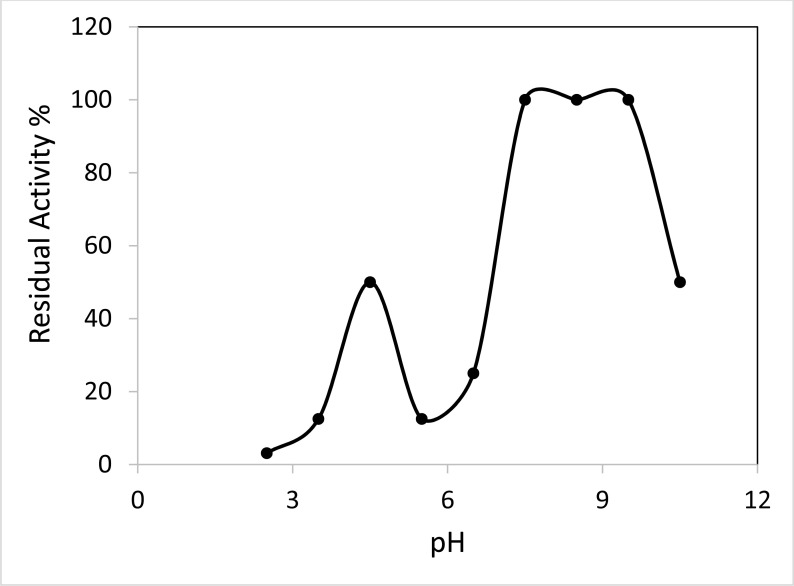
Effect of pH on ProLec hemagglutinating activity. Aliquots of lectin were incubated with buffers of different pH values ranging from 2 to 11 at room temperature for 2 hours. 0.1 N HCl or 0.1 N NaOH were used to adjust the pH of the lectin solution to neutrality. The hemagglutinating activity was assayed as shown in the experimental body.

**Figure 3B:**
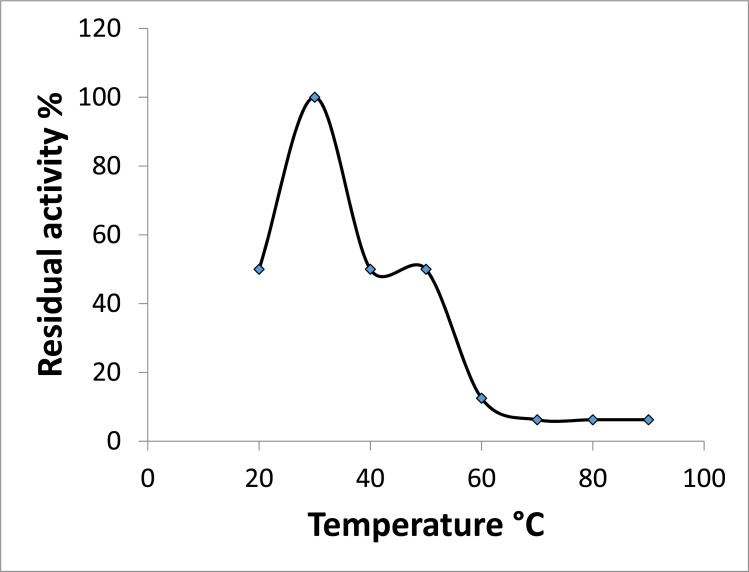
Effect of different temperature ranges on Prolec agglutinating activity. Thirty µL aliquots of lectin (1 mg/mL) were incubated in a water bath at varying temperatures ranging from 20 to 90 °C with an increment of 10 degrees for 30 minutes. The lectin samples were immediately cooled on ice and the hemagglutination activity was reported.

**Figure 3C:**
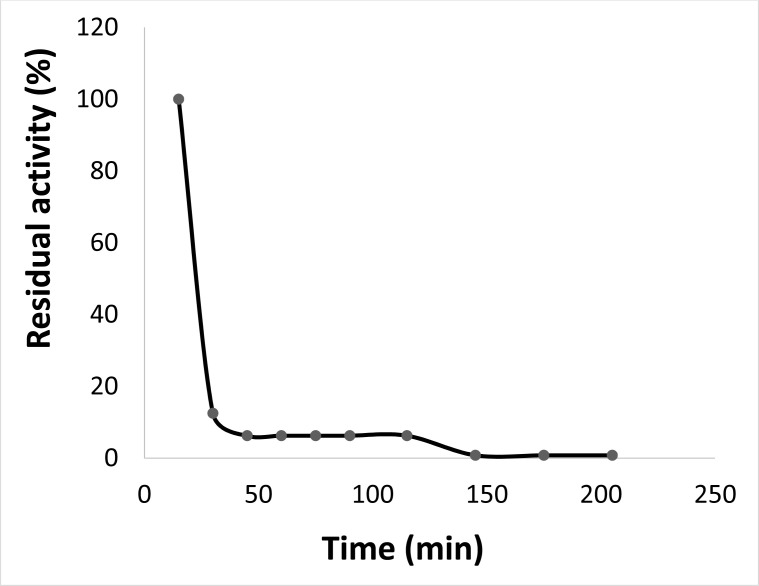
ProLec thermal stability at the optimal temperature. ProLec was incubated at its temperature optima (20 °C) for 3hrs. An aliquot was removed every 30 minutes and the hemagglutinating activity was tested as shown in the experimental body.

**Figure 7A:**
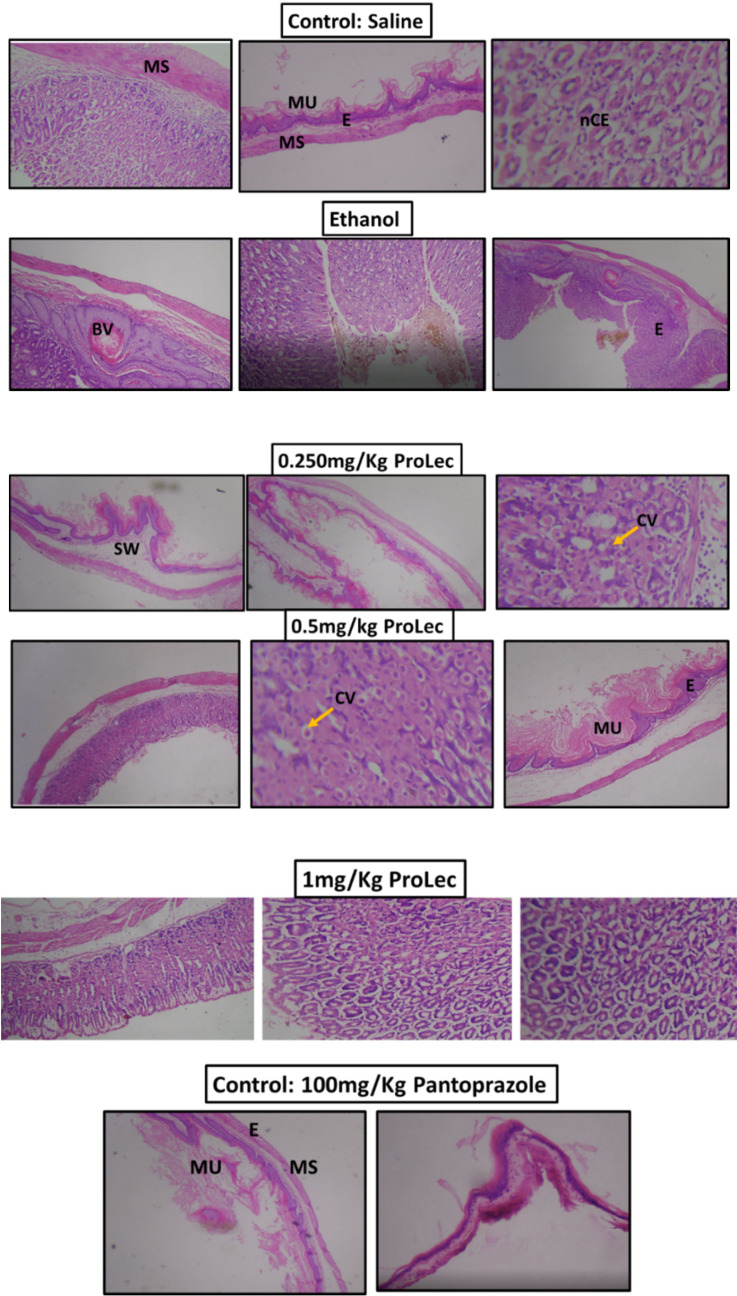
Histopathological analysis of stomach sections of different animals treated groups and controls.

**Figure 7B:**
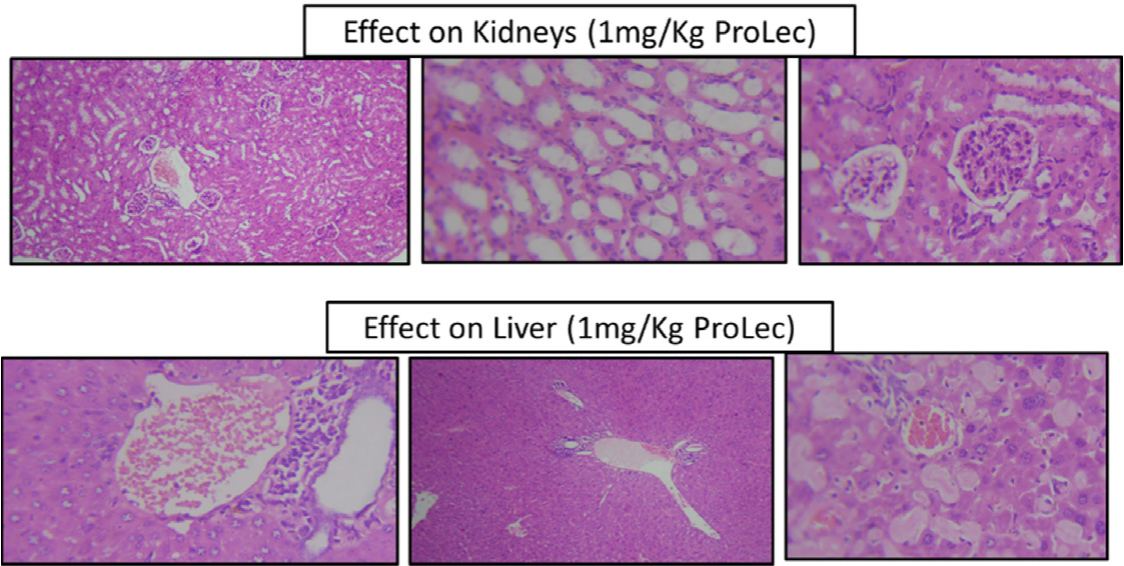
Histopathological analysis of kidneys and liver sections of rats received 1 mg/Kg ProLec.

**Table 1:** ProLec purification chart.

**Stage**	**Volume** **(mL)**	Protein Content (mg/mL)*	**Total Protein (mg)**	**Activity** **(Unit)^#^**	**Total Unit**	**Specific Activity (Unit/mg)^¥^**	**Fold Purification^£^**	**Yield (%)^€^**
Crude	400	1.5	600	16	6400	10.6	1.0	100
Affinity on Sephadex G-100	12	1.8	21.6	256	3072	142	13.4	48

**Note:** * Protein content for the crude and pure sample was estimated using Bradford assay.

# Activity Unit (titer): is the reciprocal of the highest dilution that was giving positive lectin activity.

¥ Specific activity (Unit/mg) is titer divided by mg of protein.

£ Fold of Purification is calculated by dividing the specific activity of crude by specific activity of the pure sample

€ Yield % is calculated by multiplying the obtained total Unit of the pure sample by 100 then dividing the result by the total Unit for crude extract.

**Table 2:** ProLec agglutination of human and animal erythrocytes.

**Blood Group**	**Hemagglutination Unit** **(Trypsin-Untreated erythrocytes)**	**Hemagglutination Unit** **(Trypsin-treated erythrocytes)**
**Human Erythrocytes**	2^6^	2^7^
A		
B	2^8^	2^10^
AB	2^9^	2^8^
O	2^9^	2^11^
**Animal Erythrocytes**	2^5^	2^8^
Cow		
Donkey	2^3^	2^2^
Horse	2^3^	2^2^
Camel	2^2^	2^4^
Goat	2^5^	2^9^

**Table 3:** Comparison of gastric pH mean differences between the Ethanol treated rats and the ProLec received groups (Bonferoni Posthoc analysis at P0.05).

**(I) Test Groups**	**(J) Test Groups**	**Mean Difference (I-J)**	**S.E.**	***P*-value**	**95% CI**	
Ethanol (inducer)	G1 (1mg/kg)	3.37333(*)	.67508	0.000	1.2206	5.5260
	G2 (0.5mg/kg)	3.06833(*)	.67508	0.001	.9156	5.2210
	G3 (0.25mg/kg)	2.93500(*)	.67508	0.002	.7823	5.0877
	Saline (0.9%) -ve control	3.76167(*)	.67508	0.000	1.6090	5.9144
	Pantoprazole (+ve control)	2.60333(*)	.67508	0.008	.4506	4.7560

**Note:** All groups who received lectins have a significant effect on the action of lectin in maintaining the stomach pH (P-value 0.05), the best dose is from Group 1 (Medium dose) followed by group G2 (Low dose) then group G1 (High dose)

## Data Availability

Not applicable.

## LIST OF ABBREVIATIONS

ProLec: Calotropis procera leaf lectin
HA: Hemagglutinating activity
PBS: 50 mM Phosphate buffered Saline;
N.S: Normal saline;
Tris: 50 mM Tris-HCl, pH 7.5;
Acetate: 50 mM Acetate buffer, pH 5.0
Borate: 50 mM Borate buffer, pH 9.0
BRT: 50 mM Borate buffer pH 9;
ACT: Acetate buffer pH 5.
NBS: N-bromosuccinimide
nCE: Normal cells
BV: Blood vessel
SW: Swelling
CV: Cytoplasmic vacuolization;
MU: Mucosal tissues
E: Epithelium layer
MS: Muscular layer
